# Overexpression of Reactive Oxygen Species Modulator 1 Predicts Unfavorable Clinical Outcome in EGFR-Mutant Lung Adenocarcinomas Treated With Targeted Therapy

**DOI:** 10.3389/fonc.2021.770230

**Published:** 2021-12-09

**Authors:** Won Gun Kwack, Ji-Youn Sung, Seung Hyeun Lee

**Affiliations:** ^1^ Division of Pulmonary, Allergy, and Critical Care Medicine, Department of Internal Medicine, College of Medicine, Kyung Hee University, Seoul, South Korea; ^2^ Department of Pathology, College of Medicine, Kyung Hee University, Seoul, South Korea

**Keywords:** reactive oxygen species modulator 1, biomarker, survival, epidermal growth factor receptor, targeted therapy

## Abstract

**Purpose:**

Reactive oxygen species modulator 1 (Romo1) is a novel protein that regulates the production of intracellular reactive oxygen species. Romo1 has been shown to be associated with poor survival in various clinical settings for the treatment of lung cancer. In this study, we evaluated whether tissue Romo1 expression was associated with clinical outcomes in epidermal growth factor receptor (*EGFR)*-mutated lung adenocarcinoma treated with tyrosine kinase inhibitors (TKIs).

**Method:**

Romo1 expression in tumor tissues was examined by immunohistochemistry and evaluated by histologic score. Univariate and multivariate analyses were performed to identify the clinicopathologic parameters, including Romo1 expression, which may be associated with progression-free survival (PFS), overall survival (OS), and incidence of secondary T790M mutation.

**Results:**

A total of 96 tumor specimens were analyzed. With the cut-off value of 200, 71 (74.0%) and 25 (26.0%) patients were classified into low and high Romo1 groups, respectively. The median PFS of the high Romo1 group was significantly shorter than that of the low Romo1 group (13.1 vs 19.9 months, *p* = 0.0165). The median OS of the high Romo1 group was also significantly shorter than that of the low Romo1 group (19.8 vs 37.0 months, *p* = 0.0006). Multivariate analyses showed that high Romo1 expression was independently associated with both poor PFS (hazard ratio [HR] = 2.48, 95% confidence interval [CI]: 1.35–4.56, *p* = 0.0034) and poor OS (HR = 3.17, 95% CI: 1.57–6.41, *p* = 0.0013). In addition, the rate of secondary T790M mutation after TKI failure was significantly lower in the high Romo1 group than the low Romo1 group (16.7% vs. 38.3%, *p* = 0.0369).

**Conclusions:**

Romo1 overexpression was associated with poor response to treatment and short survival in patients treated with *EGFR*-TKIs, suggesting a distinct subgroup warranting active surveillance and tailored therapeutic approach. In addition, our data highlight that Romo1 could be a potential predictive and prognostic biomarker for this patient population.

## Introduction

Lung cancer has been the leading cause of cancer-related deaths worldwide. Globally, it accounted for 2.2 million new cases and 1.8 million deaths in 2020 (1). Adenocarcinoma is the dominant histologic subtype, accounting for 60% of non-small cell lung cancers (NSCLCs) (2). Although recent advances in treatment modalities, including targeted therapy and immunotherapy, have demonstrated better clinical outcomes than conventional chemotherapy in patients with advanced lung adenocarcinoma (2, 3), the mortality of this lethal disease is still high, with a 5-year survival rate of only 24% (4), suggesting the unmet clinical need for personalized therapeutic approaches to improve prognosis.

Reactive oxygen species modulator 1 (Romo1) was first identified in 2006 in a patient with head and neck cancer who was resistant to chemotherapy after recurrence (5). Located in the mitochondrial membrane, this novel protein is a major regulator of the production of intracellular reactive oxygen species (ROS), and Romo1-induced ROS play a significant role in cell proliferation in both cancer and normal cells (6, 7). In addition, Romo1-induced ROS are related to the drug resistance of 5-fluorouracil (5-FU), and Romo1 overexpression induces invasive activity in various cancer cells (8–10). These findings suggest that Romo1 may not only be involved in carcinogenesis but may also influence the response to anticancer treatment. This concept has been proven using recent clinical data (9, 11–13). Chung et al. demonstrated that tumor Romo1 overexpression was significantly associated with poor survival and vascular invasion in patients with hepatocellular carcinoma who underwent curative resection (9). Lee et al. demonstrated that high Romo1 expression was significantly associated with early recurrence and poor survival after surgical resection in patients with NSCLC (11). Romo1 overexpression in tumor tissue was significantly associated with poor response and clinical outcomes in patients with advanced NSCLC treated with platinum doublets (12). In addition, Kim et al. reported that elevated Romo1 expression in curatively resected colorectal cancer was significantly associated with poor survival outcomes and frequent lymph node metastasis (13). These data suggest that Romo1 is a promising biomarker for malignancies. However, the clinical usefulness of this protein has never been explored in patients with cancer harboring driver genetic alterations.

In this study, we aimed to determine the clinical implications of Romo1 expression in patients with epidermal growth factor receptor (*EGFR)-*mutant lung adenocarcinoma treated with first-line tyrosine kinase inhibitors (TKIs). We investigated whether Romo1 expression is related with the response to treatment, survival rate, and the development of secondary T790M mutations after TKI failure.

## Materials and Methods

### Study Subjects and Data Collection

We retrospectively enrolled *EGFR*-mutant patients treated with first-line *EGFR*-TKIs for locally advanced or metastatic lung adenocarcinoma at Kyung Hee University Hospital, a referral hospital in South Korea, from April 2016 to March 2020. Patients without insufficient survival data, history of other malignancies, or other driving genetic alterations, including ROS proto-oncogene 1 (*ROS1)* and anaplastic lymphoma kinase (*ALK)* fusions, and those with a history of previous chemo- or radiotherapy were excluded from the study.

Staging workup was performed using chest computed tomographic (CT) scan, brain magnetic resonance imaging, and ^18^F-fluorodeoxyglucose positron emission tomography-computed tomography. Tumor-node-metastasis (TNM) staging was evaluated based on the eighth edition of the American Joint Commission on Cancer TNM staging system of NSCLC (14). After every two cycles of systemic treatment, tumor response was assessed with chest CT scan according to the Response Evaluation Criteria in Solid Tumors (RECIST) 1.1 (15). Electronic medical records were reviewed to collect patient demographics, medical or social history, and survival data. The study protocol was reviewed and approved by the Clinical Research Ethics Committee of Kyung Hee University Hospital (KHUH 2020-03-026). We obtained written informed consent from all participants who were alive. All studies were conducted in compliance with the Declaration of Helsinki.

### Romo1 Immunohistochemical Staining and Scoring

Romo1 protein expression was evaluated by immunohistochemical (IHC) staining. We used a BOND-MAX Immunoautostainer (Leica Biosystems, Newcastle, UK) of the staining after preparation of 4-micron-thick sections of paraffin-embedded tumor tissues. Antigen retrieval was performed by heating the slides at 98°C for 20 min and cooling them for 10 min in Epitope Retrieval Solution 1 and 0.01 M citrate buffer (pH 6.0), respectively. Slides were then washed in distilled water and endogenous peroxidase activity was blocked using a Bond Polymer Refine Detection Kit (Leica Biosystems, Newcastle, UK) for 5 min. After washing, the slides were placed in Tris-buffered saline and incubated for 30 min with Romo1 monoclonal antibody (OriGene Technologies, Rockville, USA) at 1:200 dilution. Subsequently, sections were developed with 3,3’-diaminobenzidine chromogen solution for 7 min, counterstained with hematoxylin, and dehydrated. Positive and negative controls were defined using human colon adenocarcinoma tissues and exclusion of the primary antibody, respectively.

Blinded to the clinical information, a pathologist (J-Y Sung) accessed the Romo1 staining intensities under a light microscope at 200 x. Stained cells were considered as positive when the cytoplasmic staining was identified. Staining intensities of individual cells were graded as 0 (no staining), 1 (weak), 2 (distinct), or 3 (strong), and the percentages of cells with these staining intensities were calculated. Finally, histological scores (H scores) were calculated by multiplying the staining intensities by percentages of cells with each staining intensity (possible range, 0–300).

### EGFR Mutation Testing

*EGFR* mutation tests were performed using tumor tissues. Genomic DNA was extracted from formalin-fixed, paraffin-embedded, 5-µm-thick tissue sections using the High Pure Template Preparation Kit (Roche Applied Science, Mannheim, Germany). The extracted DNA was stored at –20 ˚C until analysis. PANAMutyper™ (PANAGENE Inc., Daejeon, South Korea), a PNA-Clamping-based *EGFR* mutation detection kit, was used for the detection of *EGFR* mutations. The primer sets covered mutations or deletions spanning exons 18–21 of the genes encoding the tyrosine kinase domain of *EGFR*. Results were interpreted according to the manufacturer’s instructions.

### Statistical Analyses

The cut-off H score for discriminating between low and high Romo1 expression was defined as the point with the lowest *p*-value by the log-rank test for all possible H scores. Baseline characteristics of different groups were compared using the Chi-square test or Fisher’s exact test, as appropriate. Clinical outcomes including the response rate (RR), progression-free survival (PFS), and overall survival (OS) were assessed. RR was defined as the percentage of patients who achieved complete or partial response. PFS was defined as the period from the first day of treatment to disease progression or death. OS was defined as the interval from the first day of treatment to death from any cause. Data of patients without tumor recurrence or death were censored at the last follow-up. Correlations between survival outcomes and clinicopathological parameters were estimated by univariate analysis using the log-rank test, followed by Cox proportional hazard regression analysis. Parameters with *p* values < 0.1 in the univariate analysis were included for the multivariate analysis. Kaplan-Meier method was used to estimate survival rates. *p* < 0.05 was considered as statistically significant. All analyses were performed using SPSS v.20.0 (IBM Corporation, Armonk, NY, USA).

## Results

### Patient Characteristics

During the study period, 718 patients were newly diagnosed with NSCLC, and 259 patients were diagnosed with advanced lung adenocarcinoma. Of these patients, 121 received *EGFR*-TKIs as a frontline treatment for *EGFR*-positive diseases. Thirteen patients with insufficient survival data, eight with concomitant cancers, and four who received other cancer treatments before targeted therapy were excluded. Finally, 96 patients were included in the analysis.

The clinical characteristics of the study population are summarized in **Table 1**. All subjects were Korean, and their median age was 69 years (range, 41–88 years). Fifty-three (55.2%) patients were aged ≥70 years, 51 (53.1%) patients were women, and 32 (33.3%) patients were current or former smokers. Seventy-five (78.1%) patients had an Eastern Cooperative Oncology Group performance status (ECOG PS) of 0 or 1. Sixteen (16.7%) patients had stage III disease, and 80 (83.3%) patients had stage IV disease. Twenty-three (24.0%) had metastases involving three or more organs, while 22 (34.4%) and 11 (11.5%) patients had brain or liver metastases, respectively. Fifty-two (54.1%) patients had exon 19 deletion (19del), 39 (40.6%) had L858R point mutations, and 5 (5.2%) had uncommon or compound mutations. Seventy- two (74.8%) patients received afatinib, while 24 (25.2%) treated with gefitinib or erlotinib as a frontline therapy.

**Table 1:** Characteristics of 96 study patients stratified by Romo1 expression levels.

	No. of patients (%)	Romo1 H score		*p*-value
		Low (<200, n = 71)	High (≥200, n = 25)	
Age				
<70	43 (44.8)	34 (47.9)	9 (36.0)	0.3040
≥70	53 (55.2)	37 (52.1)	16 (64.0)	
Sex				
Male	45 (46.9)	32 (45.1)	13 (52.0)	0.5504
Female	51 (53.1)	39 (54.9)	12 (48.0)	
Smoking				
Never	64 (66.7)	47 (66.2)	17 (68.0)	0.8694
Ever	32 (33.3)	24 (33.8)	8 (32.0)	
Smoking intensity				
<30 pack-years	77 (80.2)	56 (78.9)	21 (84.0)	0.7722
≥30 pack-years	19 (19.8)	15 (21.1)	4 (16.0)	
ECOG PS				
0,1	75 (78.1)	55 (77.5)	20 (80.0)	0.7920
≥2	21 (21.9)	16 (22.5)	5 (20.0)	
Stage				
III	16 (16.7)	14 (19.7)	2 (8.0)	0.0747
IV	80 (83.3)	57 (80.3)	23 (92.0)	
Involved organ				
<3	73 (76.0)	53 (74.6)	20 (80.0)	0.5898
≥3	23 (24.0)	18 (25.4)	5 (20.0)	
Brain metastasis				
No	63 (65.6)	47 (66.2)	16 (64.0)	0.8423
Yes	33 (34.4)	24 (33.8)	9 (36.0)	
Liver metastasis				
No	85 (88.5)	62 (87.3)	23 (92.0)	0.7225
Yes	11 (11.5)	9 (12.7)	2 (8.0)	
*EGFR* subtypes				
19del	52 (54.1)	38 (53.5)	14 (56.0)	0.1921
L858R	39 (40.6)	28 (39.4)	11 (44.0)	
Others	5 (5.2)	5 (2.8)	0 (0.0)	
First-line TKI				0.3475
Gefitinib	16 (16.8)	10 (14.1)	6 (24.0)	
Erlotinib	8 (8.4)	4 (5.6)	4 (16.0)	
Afatinib	72 (74.8)	57 (80.3)	15 (60.0)	

ECOG PS, Eastern Cooperative Oncology Group Performance Status; EGFR, epidermal growth factor receptor; 19del, deletion mutation at exon 19; TKI, tyrosine kinase inhibitor.

### Romo1 Protein Expression

**Figure 1:**
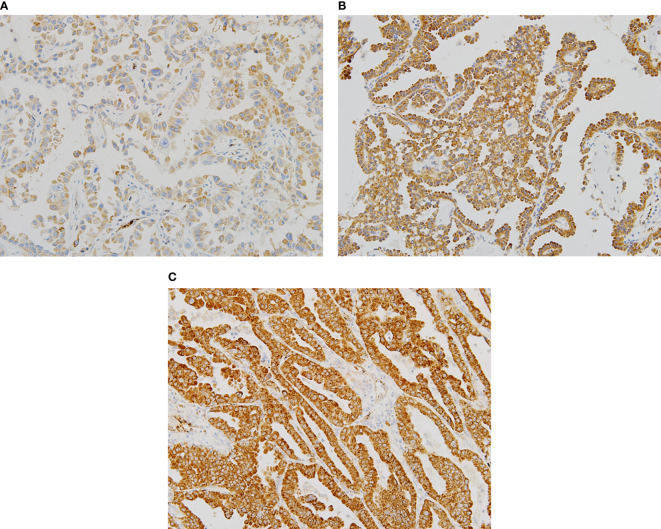
Representative examples of immunochemical staining for reactive oxygen species modulator 1 with different histologic scores (H scores) (×200). Romo1 was primarily detected in the cytoplasm. **(A)** H score of 50; **(B)** H score of 150; and **(C)** H score of 250.

Representative sections with different H scores are shown in **Figure 1**. As Romo1 is located in the mitochondrial membrane, it was primarily localized in the cytoplasm of cancer cells, as expected. The median H score of the study population was 160.0 (range, 65–290).

### Association Between Clinicopathological Parameters and Romo1 Expression

To investigate which clinicopathological parameters were associated with the level of Romo1 expression, we compared the median Romo1 H score between groups within each parameter. The median Romo1 H score was significantly higher in patients with a more advanced stage (160 vs. 135, *p* = 0.0443, **Supplementary Table 1**). We then compared the distribution of patients according to the Romo1 group. The optimal cut-off value for low and high Romo1 expression was determined to be 200 H score by the log rank test. Using this cut-off, 56 (72.7%) patients and 21 (27.3%) patients were classified to the low and high Romo1 groups, respectively. As shown in **Table 1**, Romo1 expression was not related to any parameters, including age, sex, smoking history, ECOG PS, and *EGFR* subtype. However, high Romo1 expression tended to be associated with a more advanced stage (*p* = 0.0747).

### Response Rate and PFS According to Romo1 Expression

The median follow-up period for our study subjects was 28.9 months (range, 3.1–56.0 months). The treatment response according to Romo1 expression is summarized in **Table 2**. In the low Romo1 group, one (1.4%) and 59 (83.1%) patients showed complete response (CR) and partial response (PR), respectively, while 17 (68%) patients showed PR in the high Romo1 group. The RR was significantly lower in the high Romo1 group than in the low Romo1 group (68.0% vs. 84.5%, *p* = 0.0447).

**Table 2:** Treatment response according to different Romo1 expression.

	No. of patients (%)		p-value
	Low Romo1 (n = 71)	High Romo1 (n = 25)	
Response rate (CR+PR)	60 (84.5)	17 (68.0)	0.0447
CR	1 (1.4)	0 (0)	
PR	59 (83.1)	17 (68.0)	
SD	9 (12.7)	3 (12.0)	
PD	2 (2.8)	5 (20.0)	

CR, complete response; PR, partial response; SD, stable disease; PD, progressive disease.

**Figure 2:**
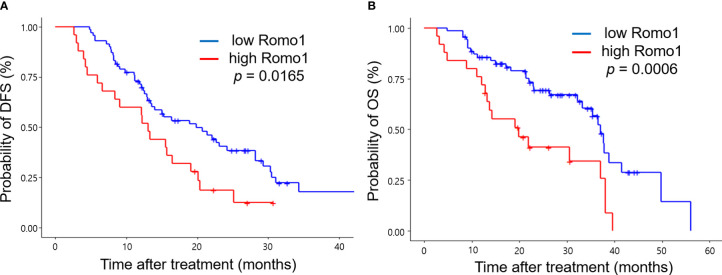
Kaplan-Meier curves of progression-free survival (PFS, **A**) and overall survival (OS, **B**) according to different expression levels of Romo1. *P*-values were determined using the log-rank test.

**Table 3** shows the PFS analysis results according to clinicopathological parameters. Seventy-seven patients (80.2%) progressed during the follow-up period. The median PFS of study population was 15.7 months (range, 2.3–33.8 months). Univariate analysis showed that male sex, heavy smoking (smoking intensity ≥30 pack-years), poor ECOG PS, involvement of three or more organs, and presence of liver metastasis were associated with poor PFS (all *p* < 0.05). In addition, high Romo1 expression was significantly associated with shorter PFS (*p=*0.0165). Multivariate analysis showed that male sex (hazard ratio [HR] = 2.09, 95% confidence interval [CI]: 1.08–4.03), heavy smoking (HR = 2.13, 95% CI: 1.02–4.45), poor ECOG PS (HR = 2.97, 95% CI: 1.53–5.75), presence of liver metastasis (HR = 4.25, 95% CI: 1.85–9.76), and high Romo1 (HR = 2.48, 95% CI: 1.35–4.56) were independently associated with shorter PFS. Patients with high Romo1 expression were likely to have poor PFS compared to those with low Romo1 expression as shown in Kaplan–Meier survival curves (**Figure 2A**).

**Table 3:** Progression-free survival according to clinicopathological parameters of all study subjects (n=96).

	Median PFS (months)	Univariate		Multivariate	
		HR (95% CI)	*p-*value	HR (95% CI)	*p*-value
All	15.7				
Age			0.2190	NA	
<70	15.7	reference			
≥70	15.0	1.36 (0.83-2.22)			
Sex			0.0050		0.0288
Male	12.6	2.00 (1.23-3.24)		2.09 (1.08-4.03)	
Female	22.1	reference		reference	
Smoking history			0.0933		0.4170
Never	19.9	reference		1.39 (0.68-3.14)	
Ever	13.0	1.54 (0.93-2.54)		reference	
Smoking intensity			0.0210		0.0450
<30 pack-years	19.9	reference		reference	
≥30 pack-years	11.2	1.95 (1.11-3.45)		2.13 (1.02-4.45)	
ECOG PS			0.0204		0.0013
0, 1	20.1	reference		reference	
≥2	12.1	1.92 (1.11-3.32)		2.97 (1.53-5.75)	
Stage			0.1050		0.5860
III	29.8	0.55 (0.27-1.13)		1.27 (0.54-3.01)	
IV	15.5	reference		reference	
Involved organ			0.0188		
<3	19.9	reference		reference	
≥3	12.4	1.90 (1.11-3.24)		1.29 (0.65-2.56)	0.4622
Brain metastasis			0.8982	NA	
No	16.4	reference			
Yes	15.7	1.03 (0.62-1.71)			
Liver metastasis			0.0061		0.0006
No	19.9	reference		reference	
Yes	12.1	2.56 (1.31-5.00)		4.25 (1.85-9.76)	
EGFR subtypes*			0.2850	NA	
19del	15.7	1.39 (0.15-2.85)			
L858R	15.3	reference			
First-line TKI			0.0821		0.1208
Gefitinib/erlotinib	13.3	1.98 (1.03-4.16)		1.45 (0.88-7.10)	
Afatinib	18.5	reference		reference	
Romo1 expression			0.0165		0.0034
Low (<200)	19.9	reference		reference	
High (≥200)	13.1	1.90 (1.13-3.22)		2.48 (1.35-4.56)	

*Analysis of 91 patients, excluding 5 patients with uncommon or compound mutations.

ECOG PS, Eastern Cooperative Oncology Group Performance Status; EGFR, epidermal growth factor receptor; 19del, deletion mutation at exon 19; TKI, tyrosine kinase inhibitor; HR, hazard ratio; CI, confidence interval; NA, not analyzed.

### OS According to Romo1 Expression

**Table 4** shows the results of the OS analysis. The median OS of study population was 36.5 months (range, 3.5–52.8 months). Univariate analysis showed that heavy smoking, metastases involving three or more organs, and presence of liver metastasis were significantly associated with shorter OS (all *p* < 0.05). In addition, high Romo1 expression was significantly associated with shorter OS (*p* = 0.0006). Multivariate analysis showed that heavy smoking (HR = 2.77, 95% CI: 1.31–5.70), presence of liver metastasis (HR = 2.97, 95% CI: 1.24–7.14), and high Romo1 expression (HR = 3.17, 95% CI: 1.57–6.41) were independently associated with shorter OS. Patients with high Romo1 expression were likely to have poor OS compared to those with low Romo1 expression as shown in Kaplan–Meier survival curves (**Figure 2B**).

**Table 4:** Overall survival according to clinicopathological parameters of all study subjects (n=96).

	Median OS (months)	Univariate		Multivariate	
		HR (95% CI)	*p-*value	HR (95% CI)	*p-value
All	36.5				
Age				NA	
<70	36.5	reference			
≥70	35.4	1.42 (0.8-2.51)	0.2321		
Sex					0.5032
Male	32.2	1.60 (0.89-2.87)	0.1147	1.31 (0.59-2.89)	
Female	37.1	reference		reference	
Smoking History			0.0750		0.0619
Never	37.1	reference		reference	
Ever	32.2	1.71 (0.95-3.10)		2.22 (0.96-5.1)	
Smoking intensity			0.0068		0.0157
<30 pack-years	37.0	reference		reference	
≥30 pack-years	32.2	2.31 (1.26-4.24)		2.77 (1.31-5.70)	
ECOG PS			0.4443	NA	
0, 1	36.5	reference			
≥2	33.2	1.28 (0.68-2.4)			
					
Stage			0.8138	NA	
III	30.5	reference			
IV	33.2	1.09 (0.52-2.28)			
Involved organ			0.0048		0.0862
<3	37.1	reference		reference	
≥3	21.4	2.41 (1.31-4.44)		2.00 (0.91-4.41)	
Brain metastasis			0.9999	NA	
No	36.5	reference			
Yes	35.4	1.04 (0.56-1.79)			
Liver metastasis			0.0008		0.0148
No	36.5	reference		reference	
Yes	22.0	2.93 (1.57-5.51)		2.97 (1.24-7.16)	
EGFR subtypes*			0.7684	NA	
19del	37.0	1.23 (0.32-4.73)			
L858R	33.2	reference			
First-line TKIs					0.2019
Gefitinib/erlotinib	30.2	1.70 (0.95-3.57)	0.0847	1.41 (0.69-3.27)	
Afatinib	36.1	reference		reference	
Romo1 expression			0.0006		0.0013
Low (<200)	37.0	reference		reference	
High (≥200)	19.8	2.77 (1.55-4.96)		3.17 (1.57-6.41)	

*Analysis for 91 patients excluding 5 patients with uncommon or compound mutations.

ECOG PS, Eastern Cooperative Oncology Group Performance Status; EGFR, epidermal growth factor receptor; 19del, deletion mutation at exon 19; TKI, tyrosine kinase inhibitor; HR, hazard ratio; CI, confidence interval; NA, not analysed.

### Frequency of Acquired T790M Mutation According to Romo1 Expression

**Figure 3:**
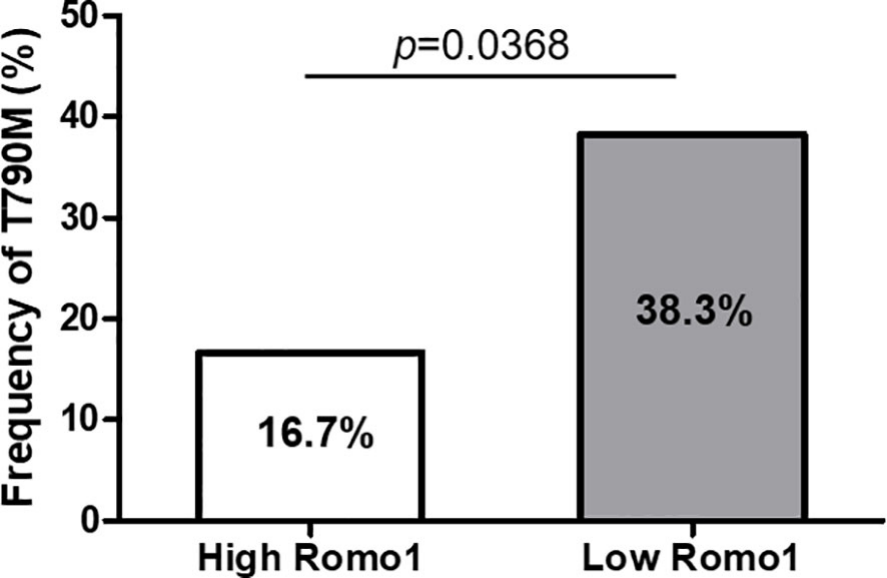
Frequency of secondary T790M mutation after tyrosine kinase inhibitor (TKI) failure according to different expression levels of Romo1. The rate of T790M mutation is significantly lower in the high Romo1 group compared with the low Romo1 group (*p* = 0.0369).

To identify whether Romo1 expression is associated with the emergence of a secondary T790M mutation, we evaluated the mutation rates according to different Romo1 groups. Among 77 patients who progressed after first-line TKI therapy, 56 (72.7%) underwent T790M mutation testing. The frequencies of T790M stratified by clinicopathological parameters of the 56 patients are summarized in **Table 5**. Overall, 20 (20/56, 35.7%) patients harbored the T790M mutation. The mutation rates were significantly lower in patients with the L858R mutation and those who received TKI for less than 12 months (all *p*< 0.05). In addition, patients with high Romo1 expression showed a significantly lower frequency of T790M mutation than those with low Romo1 expression (16.7% vs. 38.3%, *p* = 0.0365; **Figure 3**). Univariate analysis for the factors associated with the frequency of T790M mutation showed that L858R mutation, TKI use for less than 12 months, and high Romo1 expression were significantly associated with a lower incidence of T790M mutations (all *p* < 0.05). Multivariate analysis showed that the L858R mutation (odds ratio [OR] = 0.46, 95% CI: 0.08–0.94), TKI use for less than 12 months (OR = 0.23, 95% CI: 0.05–0.89), and high Romo1 expression (OR = 0.36, 95% CI: 0.07–0.96) were independently associated with a low incidence of acquired T790M.

**Table 5:** Analysis for the factors associated with emergence of secondary T790M mutation (n=56).

	T790M		*p-*value*	Univariate analysis		Multivariate analysis	
	Negative (n = 36)	Positive (n = 20)		OR (95% CI)	p-value	OR (95% CI)	*p-*value
All							
Age			0.0987		0.0926		0.1368
<70	15 (55.6)	12 (44.4)		0.54 (0.12 -1.31)		0.35 (0.17-1.92)	
≥70	21 (75.0)	8 (25.0)		reference		reference	
Sex			0.6895		0.6897	NA	
Male	19 (65.5)	10 (34.5)		reference			
Female	17 (63.0)	10 (27.0)		0.81 (0.28 -2.30)			
Smoking History			0.0781		0.0857		0.0565
Never	19 (54.3)	16 (45.7)		0.34 (0.10 -1.17)		0.23 (0.05-1.04)	
Ever	17 (80.9)	4 (19.1)		reference		reference	
Smoking intensity			0.3558		0.2735	NA	
<30 pack-years	26 (60.5)	17 (39.5)		0.46 (0.12 -1.84)			
≥30 pack-years	10 (79.6)	3 (20.4)		reference			
ECOG PS			0.1136		0.0989		0.1105
0, 1	28 (70.0)	12 (30.0)		reference		reference	
≥2	8 (50.0)	8 (50.0)		0.41 (0.13-1.26)		0.34 (0.09-1.28)	
Stage			0.2458		0.8063	NA	
III	4 (57.1)	3 (42.9)		0.83 (0.19 -3.71)			
IV	31 (64.6)	17 (35.4)		reference			
Involved organ			0.9861		0.9861	NA	
<3	25 (64.1)	14 (35.9)		reference			
≥3	11 (64.7)	6 (35.3)		0.99 (0.32 -3.10)			
Brain metastasis			0.2301		0.2333	NA	
No	25 (69.4)	11 (30.6)		reference			
Yes	11 (22.0)	9 (78.0)		0.52 (0.18-1.53)			
Liver metastasis			0.7210		0.6068	NA	
No	30 (65.2)	16 (34.8)		reference			
Yes	6 (60.0)	4 (40.0)		0.70 (0.18 -2.72)			
EGFR subtypes			0.0460		0.0339		0.0413
19del	11 (52.3)	10 (47.7)		reference		reference	
L858R	25 (75.7)	8 (24.3)		0.29 (0.09-0.91)		0.46 (0.08-0.94)	
First-line TKI*			0.0846		0.0629		0.2614
Gefitinib/erlotinib	11 (55.0)	8 (45.0)		reference		reference	
Afatinib	24 (66.7)	12 (33.3)		0.50 (0.21-1.78)		0.65 (0.12-1.39)	
Duration of TKI use			0.0318		0.0457		0.0411
<12 months	17 (80.9)	4 (19.1)		0.24 (0.10 -0.91)		0.23 (0.05-0.89)	
≥12months	20 (57.1)	15 (42.9)		reference		reference	
Romo1 expression			0.0365		0.0401		0.0459
Low (<200)	22 (61.1)	17 (38.3)		0.28 (0.07-0.98)		0.36 (0.07-0.96)	
High (≥200)	14 (82.3.)	3 (16.7)		reference		reference	

*Analysis after excluding 2 patients with uncommon or compound mutations.

ECOG PS, Eastern Cooperative Oncology Group Performance Status; EGFR, epidermal growth factor receptor; 19del, deletion mutation at exon 19; TKI, tyrosine kinase inhibitor; OR, odds ratio; CI, confidence interval; NA, not analysed.

## Discussion

Our study demonstrated that high Romo1 expression was significantly associated with poor PFS and OS in patients with *EGFR*-mutant lung adenocarcinoma treated with frontline targeted therapy. In addition, it was associated with lower rate of secondary T790M mutations after TKI use. To the best of our knowledge, this is the first study to suggest the predictive and prognostic value of Romo1 in oncogene-driven malignancies. The current data highlight that high Romo1 expression might confer an aggressive phenotype, requiring tailored therapeutic approaches among *EGFR*-mutant tumors.

*EGFR* mutations are major driving genetic alterations that account for 15–50% of patients diagnosed with lung adenocarcinoma (14, 15). *EGFR*-TKIs are recommended as the first-line treatment in patients harboring these mutations as they improve both PFS and OS compared with those only treated with chemotherapy (16). However, the efficacy of *EGFR*-TKIs is still suboptimal, mainly because of the primary and secondary resistance, which is one of the major challenges in the management of these patients (17–19). Identification of the mechanisms leading to TKI resistance or prediction of treatment response before TKI use is critical to improve the clinical outcomes in this patient population. Although the resistant mechanism for *EGFR*-TKIs has been widely studied, it is not fully understood; T790M mutation, concurrent genetic alterations, *MET* amplification, epithelial-mesenchymal transition (EMT), and histological transformation are considered to be contributing factors (16, 17).

Cancer cells are characterized by increased ROS levels compared with normal cells, and the upregulated ROS are involved in various biological processes of carcinogenesis, including cell signaling, proliferation, and metastasis, as well as chemoresistance (18–20). Cumulating evidence indicates that ROS play a critical role in *EGFR*-TKI resistance. Increased oxidative stress induces TKI resistance of *EGFR*-mutant cells by activating phosphorylation and impairing the dimerization of *EGFR* (21, 22). In addition, ROS play a critical role in EMT engagement (23, 24) and interferon gamma-induced Src activation *via* the Raf/ERK signaling pathway (25). Moreover, chronic treatment with *EGFR*-TKIs enhances intracellular oxidative stress through NOX4, and morphologic changes and dysfunction of mitochondria (26, 27). Antioxidants can alleviate TKI-resistance by inhibiting ROS-induced aberrant phosphorylation of EGFR and AKT (26, 28). Taken together, ROS are involved in both primary and secondary resistance to *EGFR*-TKIs *via* various mechanisms, including abnormal *EGFR* phosphorylation, EMT, and Sarc activation.

Romo1 is a major regulator of intracellular ROS production, and Romo1-induced ROS are indispensable for the proliferation of normal and cancer cells (6, 7). Studies have consistently demonstrated the potential value of Romo1 as a novel biomarker for various lung cancer treatment (11, 12, 29, 30). **Table 6** summarizes these previous studies and the present data, evaluating the clinical implications of Romo1 in lung cancer patients. Different studies have demonstrated different cutoffs from each other; however, the low H score cutoffs in two studies (11, 30) can be attributed to the early stage population enrolled in those studies. The present data suggest that Romo1 levels seem to be abundant in advanced disease. Based on the critical association between Romo1 and ROS production and the contribution of ROS in *EGFR*-TKI resistance, we hypothesized that Romo1 may be a novel biomarker in patients treated with targeted therapy. In addition to the predictive and prognostic values of Romo1, our present data suggest the possibility of this novel protein as a potential treatment target for *EGFR*-mutant NSCLC, considering the crucial association between ROS and TKI resistance. To address this issue, we have just started a preclinical study on the regulation mechanism of Romo1, combinational effect of Romo1 inhibition and *EGFR*-TKIs, and drug screening for its inhibitors.

**Table 6:** Summary of studies on the predictive and prognostic value of Romo1 in lung cancer.

Author, year	Patients number	Treatment setting	Sample used	Quantification method	Cut-off H score	Association with Romo1 expression
Lee et al., 2015 (11)	110	Surgery	Tissue	IHC with H score	159	DFS, OS
Lee et al., 2017 (12)	88	Chemotherapy	Tissue	IHC with H score	200	PFS, OS
Kong et al., 2019 (29)	49	Definitive radiotherapy	Tissue	IHC with H score	200	PFS, OS
Kong et al., 2020 (30)	40	Stereotactic radiosurgery	Tissue	IHC with H score	125	DMFS
The present study	96	EGFR-TKI	Tissue	IHC with H score	200	PFS, OS, frequency of T790M mutation

IHC, immunohistochemistry; H score, Histologic score; DFS, disease-free survival; PFS, progression-free survival; OS, overall survival; EGFR, epidermal growth factor receptor; TKI, tyrosine kinase inhibitor; DMFS, Distant metastasis-free survival.

Recent studies have demonstrated the clinical usefulness of Romo1 gene expression and the novel association between micro RNA and Romo1. Alizamir et al. demonstrated that Romo1 mRNA expression increased in bladder cancer tissues and that Romo1 overexpression was associated with advanced tumor (31). Similarly, Afshar et al. showed that Romo1 mRNA was overexpressed in gastric cancer tissues and that its overexpression was associated with unfavorable prognosis in patients with gastric cancer (32). Moreover, Wang et al. reported that LINC00319, a long non coding RNA, is correlated with early recurrence of bladder cancer, and that LINC00319 is a sponge for miR-4492, which directly targets Romo1 expression. In that study, the miR-4492/Romo1 axis was shown to regulate proliferation, migration, and tumor invasion of bladder cancer cells (33). These findings suggest that evaluating Romo1 gene expression could be more useful than evaluating protein expression using IHC because the former can be less variable than the latter. In addition, the potential role of micro RNA as a regulator of Romo1 in various human malignancies should be investigated in future studies.

Secondary T790M mutation is a major resistance mechanism and has been identified in approximately half of patients who progressed after *EGFR*-TKI therapy (34). Detection of this mutation is important for the management of *EGFR*-mutant patients because T790M-positive tumors are susceptible to third-generation *EGFR*-TKIs, such as osimertinib. Although mutational analysis using tumor tissue or body fluid is recommended, it is often limited by factors, such as the invasiveness of tissue acquisition and the insufficiency or inaccessibility of tissue samples. Previous studies have demonstrated that the frequent T790M mutation is associated with 19del and long-term exposure to *EGFR*-TKIs (35–37). Our present data confirmed these previous findings, and additionally suggest that high Romo1 expression could be a negative predictor of T790M development after frontline TKI. The reason that Romo1 is associated with the emergence of the T790M mutation is unclear; however, it may be attributed to the different durations of treatment with *EGFR*-TKI according to different Romo1 expression. As suggested in the previous studies, T790M-positive cells undergo selection and are enriched during *EGFR*-TKI treatment (38–40). In addition, time is required for the development of T790M-dominant tumor because cells harboring the mutation grow slowly compared with T790M-negative counterparts (41). Based on these findings, we hypothesize that shorter treatment duration may be the cause of lower frequency of the T790M mutation in high Romo1 population. Our hypothesis should be elucidated through further investigations.

The present data suggest that Romo1 overexpression confers a distinct aggressive phenotype that may require different treatment strategies. Because TKI monotherapy could be less effective in patients with high Romo1 expression, other therapeutic options, such as combination with chemotherapy or immunotherapy, can be considered. A recent study showed that pemetrexed-based doublet combined with gefitinib showed better RR (84% vs. 67%, *p* < 0.001), longer PFS (20.9 vs. 11.9 months, HR = 0.49; *p* < 0.001), and OS (50.9 vs. 38.8 months, HR = 0.722; *p* = 0.021) compared with gefitinib monotherapy in *EGFR*-mutant tumors (42). To prove our hypothesis and determine the optimal treatment strategy for those with different Romo1 expression levels, large-scale prospective investigations are required.

This study had several limitations. First, it is a relatively small retrospective study performed at a single institute, and the slides were reviewed by a single pathologist. Second, we did not include nutritional status or comorbidities as clinical characteristics, which might influence the survival outcome of our patient population. To overcome these potential caveats, we aimed to enhance the validity of our data by long-term follow-up. Third, tumors harboring other driver genetic alterations, such as *ROS1* and *ALK* fusions, were excluded because of their low frequency in our patient cohort. Fourth, the interval changes in Romo1 expression in patients with progressive disease after TKI failure were not investigated. As mentioned earlier, *EGFR*-TKIs can induce ROS production, and thus, Romo1 expression can be affected by previous treatments. The predicted value or impact of the dynamics of Romo1 expression on subsequent treatment could be an interesting topic for future studies. Finally, we did not simultaneously explore other factors that could be associated with TKI resistance, including immune phenotypes or co-existing genetic alterations. We are currently working on a possible association between Romo1 expression and immunologic signatures or concurrent genetic alterations in *EGFR*-mutant populations.

## Conclusion

The present data demonstrate that high Romo1 expression is associated with unfavorable clinical outcomes, not only due to the poor response to first-line TKI treatment but also because of the less emergence of secondary T790M mutation. These findings highlight that Romo1 overexpression might confer a distinct aggressive phenotype among *EGFR*-mutant tumors, requiring a different therapeutic approach, although further studies are needed to verify our results. In addition, future studies should focus on whether Romo1 has similar clinical implications in other malignancies harboring driver genetic alterations, and optimal treatment strategy for the Romo1-overexpressed, *EGFR*-mutant tumors. Large prospective studies validating our data might facilitate the clinical use of Romo1 expression for the risk stratification and prediction of prognosis in those patient population.

## Data Availability Statement

The original contributions presented in the study are included in the article/**Supplementary Material**. Further inquiries can be directed to the corresponding author.

## Ethics Statement

The studies involving human participants were reviewed and approved by Kyung Hee University Hospital. The patients/participants provided their written informed consent to participate in this study.

## Author Contributions

SHL conceived and designed the study. WGK, J-YS, and SHL collected, analyzed, and interpreted the data, and drafted the article. SHL revised the paper critically. All authors contributed to the article and approved the submitted version.

## Funding

This research was supported by a grant from the Basic Research Program through the National Research Foundation funded by the Ministry of Science and ICT (2019R1F1A1041812), and a grant of the Korea Health Technology R&D Project through the Korea Health Industry Development Institute (KHIDI) funded by the Ministry of Health and Welfare (HI18C1944) of the Republic of Korea.

## Conflict of Interest

The authors declare that the research was conducted in the absence of any commercial or financial relationships that could be construed as a potential conflict of interest.

## Publisher’s Note

All claims expressed in this article are solely those of the authors and do not necessarily represent those of their affiliated organizations, or those of the publisher, the editors and the reviewers. Any product that may be evaluated in this article, or claim that may be made by its manufacturer, is not guaranteed or endorsed by the publisher.
