# Supplementary material for: Overexpression of Reactive Oxygen Species Modulator 1 Predicts Unfavorable Clinical Outcome in EGFR-Mutant Lung Adenocarcinomas Treated With Targeted Therapy
Source: Front Oncol. 2021 Dec 9;11:770230. doi: 10.3389/fonc.2021.770230 (PMC8695430; doi:10.3389/fonc.2021.770230)
Supplement: Supplementary file 1 [file Table_1.docx]

Supplementary Material

**Supplementary Table 1** Romo1 expression according to different clinicopathological parameters

|  | No. of patients (%) | Romo1 H score* | *p*-value |
| --- | --- | --- | --- |
| All | 96 (100.0) | 160 (110-200) |  |
| Age |  |  | 0.1686 |
| <70 | 43 (44.8) | 150 (110-180) |  |
| ≥70 | 53 (55.2) | 175 (110-200) |  |
| Sex |  |  | 0.7031 |
| Male | 45 (46.9) | 160 (110-200) |  |
| Female | 51 (53.1) | 150 (110-195) |  |
| Smoking |  |  | 0.4709 |
| Never | 64 (66.7) | 160 (120-200) |  |
| Ever | 32 (33.3) | 155 (110-195) |  |
| Smoking intensity |  |  | 0.2660 |
| <30 pack-years | 77 (80.2) | 160 (120-200) |  |
| ≥30 pack-years | 19 (19.8) | 150 (110-190) |  |
| ECOG PS |  |  | 0.3461 |
| 0,1 | 75 (78.1) | 160 (120-200) |  |
| ≥2 | 21 (21.9) | 150 (110-200) |  |
| Stage |  |  | 0.0443 |
| III | 16 (16.7) | 135 (95-165) |  |
| IV | 80 (83.3) | 160 (120-200) |  |
| Involved organ |  |  | 0.4461 |
| <3 | 73 (76.0) | 160 (120-200) |  |
| ≥3 | 23 (24.0) | 125 (105-195) |  |
| Brain metastasis |  |  | 0.6496 |
| No | 63 (65.6) | 160 (120-200) |  |
| Yes | 33 (34.4) | 150 (110-200) |  |
| Liver metastasis |  |  | 0.5167 |
| No | 85 (88.5) | 160 (110-200) |  |
| Yes | 11 (11.5) | 150 (100-190) |  |
| ECOG PS |  |  | 0.3537 |
| 0,1 | 75 (78.1) | 160 (110-200) |  |
| ≥2 | 21 (21.9) | 120 (110-195) |  |
| *EGFR* subtypes |  |  | 0.4830 |
| 19del | 52 (54.1) | 160 (120-215) |  |
| L858R | 39 (40.6) | 140 (110-180) |  |
| Others | 5 (5.2) | 150 (90-150) |  |
| First-line TKI |  |  | 0.2537 |
| Gefitinib | 16 (16.8) | 160 (120-215) |  |
| Erlotinib | 8 (8.4) | 135 (110-190) |  |
| Afatinib | 72 (74.8) | 145 (100-180) |  |

*presented as median (interquartile range)

ECOG PS, Eastern Cooperative Oncology Group Performance Status; EGFR, epidermal growth factor receptor; 19del, deletion mutation at exon 19; TKI, tyrosine kinase inhibitor
